# Stewardship of Wild and Farmed Edible Insects as Food and Feed in Sub-Saharan Africa: A Perspective

**DOI:** 10.3389/fvets.2021.601386

**Published:** 2021-02-19

**Authors:** Robert Musundire, Dianah Ngonyama, Abel Chemura, Ruth Tambudzai Ngadze, Jose Jackson, Margaret Jekanyika Matanda, Tawanda Tarakini, Maud Langton, Linley Chiwona-Karltun

**Affiliations:** ^1^Department of Crop Science and Post-Harvest Technology, Chinhoyi University of Technology, Chinhoyi, Zimbabwe; ^2^Association of African Agricultural Professionals in the Diaspora (AAAPD), Des Moines, IA, United States; ^3^Department of Environmental Science, Chinhoyi University of Technology, Chinhoyi, Zimbabwe; ^4^Department of Food Science and Technology, Chinhoyi University of Technology, Chinhoyi, Zimbabwe; ^5^Alliance for African Partnership, Michigan State University, East Lansing, MI, United States; ^6^The University of Sydney, Sydney, NSW, Australia; ^7^Department of Wildlife Ecology and Conservation, Chinhoyi University of Technology, Chinhoyi, Zimbabwe; ^8^Department of Molecular Sciences, Swedish University of Agricultural Sciences, Uppsala, Sweden; ^9^Department of Urban and Rural Development, Swedish University of Agricultural Sciences, Uppsala, Sweden

**Keywords:** edible insects, policy, Sub Saharan Africa, stewardship, sustainability, women

## Abstract

Edible insects have gained popularity as alternative food resources in the face of climate change and increasing carbon and environmental footprints associated with conventional agricultural production. Among the positive attributes that make edible insects suitable as food and feed substrates include rapid reproduction, high energy conversion efficiency, wide distribution, diversity, reduced greenhouses gases and ammonia emissions, possibility to reduce waste and high nutritional composition. In Sub-Saharan Africa, considerable scientific data exist on use of insects as food and livestock feed. However, coherent policies regarding safety, sustainability, trade and regulation of insects as food and animal feed are lacking. The benefits associated with edible insects are likely to accrue in Sub-Saharan Africa through use of a combination of approaches such as ensured sustainable utilization of edible insects in the wild, preservation of traditional conservation, harvesting and consumption practices, development of captive mass production schemes and strengthening robust value chains to incentivise indigenous participants. Collectively these approaches are referred to as the steward and use of insects as food and animal feed. This paper examines the policy frameworks that exist to support the use of edible insects as food and feed on the African continent. This investigation employed a literature review focussing on national policies in selected African countries to assess the relevance to edible insects. Using a baseline of more than 10 edible insect species consumed, 10 country cases in Sub-Saharan Africa were used to support our in-depth examination of the policy situation that may support good stewardship of edible insects as food and feed. Focus on how policies encompassing biodiversity, natural resources, culture, education, research, technology development, trade, health and nutrition and how that could be improved to support inclusivity of edible insects is discussed. We conclude by proposing a pathway that may accelerate recognition and valorisation of edible insects as important food and feed resources in Sub-Saharan Africa including improving policies to support good stewardship of these resources for sustainability.

## Introduction

Several studies in the 1970s documented the diversity of edible insect species consumed by indigenous people in different parts of Africa. Most of this literature before the dawn of the new millennium focused on documenting traditional insect harvesting and consumption practices (1–6). However, in the recent years, a number of researchers have taken further steps to provide additional data on the identification of edible insects, determining insects' nutritional composition, harvesting, and preparation methods for insect consumption (7–14).

While there have been increased efforts in Sub-Saharan Africa (SSA) to suppport research and development through funding and setting up of institutions, the policy environment in this region is still riddled with lack of (or fragmented and non-coherent) policies and frameworks that support and promote the sustainable utilization of insects as food or feed. Niassy et al. (15) assesed the policy environment in relation to the use of insects as food and feed in South Africa and concluded that while there was a conducive policy environment for promotion of edible insects, there was a general lack of national policy frameworks to intergratively utilize insects as food in a coordinated way. The main findings by Grabowski et al. (16) through an extensive study on legislation edible insects in Africa indicate that insects are not mentioned in national regulations in relation to their use as food. Additionally, this study highlighted minimal existence of legal instruments to consider insects as food, and yet insects have been consumed as food for decades in Africa This conclusion also applies to several Sub-Saharan African countries where government legislative frameworks scantly give reference to sustainable utilization of edible insects as a main priority.

With respect to wild harvested edible insect species, there is increasing population pressure on forest resources. In southern Africa, scientific studies over the past decade have all pointed to the diminishing population of the widely consumed mopane worm (*Imbrasia belina)*. Gondo et al. (17) attributed declining populations in southern Zimbabwe of mopane worms to increased commercialization and overexploitation. In south Africa, Baiyegunhi et al. (18) and Sekonya et al. (19) attribute lack of institutions and laws to regulate use of mopane worms as the major drivers affecting biodiversity. This is exacerbated by continuous demand driven harvesting to the detriment of wild populations. Changing land use patterns have also been shown to negatively affect wild insect population abundances and harvests due to conversion of land into agriculture, deforestation and decline in vegetation cover (20). Climate change driven environmental shifts also demand that we safeguard traditional harvesting and consumption practices of edible insects in several parts of Africa in order to ensure continued availability of these resources for future generations. Similarly, a need to safeguard mordern technologies of insect farming also arises with increased pressure to support traditional harvesting in order to ensure minimal negative effects to the environment. Collectively in the modern context there is need for a combined effort by governments in Sub-Sahara Africa to ensure positive integration of insects as food and feed into the current food systems as well as in main stream economic activities in the region.

In an environment beset with slowed economic development and reduced investment oppportunities, it can be prudent to recognize that continued use of edible insects as sustainable nutritious food resources in the short to medium term would still rely to a larger extent on wild harvested insect species. Regulation or stewardship in the use of edible insects as food resources has great application especially in Sub-Saharan Africa. The term stewardship as defined by Worrell and Appleby (21) *as the responsible use of natural resources in the best interests of society taking into consideration current and future needs*. In a wide range of disciplines, such as marine resources, environmental and pesticide use management, stewardship has been a common philosophy applied in circumstances where collective, responsible actions and accountability are necessary for posterity (21–23). When applied to the use of insects as food and feed, this term captures aspects of sustainability, environmental risk and policy legislation (24).

This paper reviews case studies in selected countries in Sub-Saharan Africa regarding the situational and policy environment related to edible insects. Furthermore, the paper gives a perspective on how current traditional insect consumption practices can be structured and integrated into institutional, national, regional and continental policies in Africa. We conclude by providing a theoretical framework for good stewardship of edible insects. To the best of our knowledge previous reviews on legislation of the edible insect sector in Africa have mainly interrogated the legal status of insects as food (16) and have not extensively discussed an African integrated system that can facilitate good stewardship in the use of edible insects.

## Is There Evidence for Good Stewardship of Edible Insects' Utilization in SSA?

### Evidence From Harvesting Methods From the Wild

The bulk of edible insects consumed in Africa are currently harvested or gathered based on seasonal natural occurrences in wild and cultivated habitats (8, 14, 25–30). Over many years, indigenous African insect consumers have mastered skills of harvesting edible insects from the wild. Chavhunduka (2) documented several traditional methods used for harvesting edible insects from the wild in Zimbabwe while van Huis (31) and DeFoliart (32) recorded traditional approaches used in different parts of Africa. Documented records provide evidence that most of the traditional harvesting methods are based on knowledge of insect life-history strategies (such as patterns of survival, feeding and reproduction) by local gatherers. Records also indicate that indigenous communities utilize methods that facilitate efficient collection of insects while taking into consideration the need to sustain subsequent insect generations (10, 14). In the Central African Republic, Vantomme et al. (33) reported a coordinated approach of conserving the caterpillar species (*Imbrasia oyemensis)* by conserving the sapelli tree (*Entandrophragma cylindricum)* per given unit area during forest logging. In Zambia, Holden (34) reported a reduction in bush fires associated with the need to protect wild harvested insects.

**Figure 1:**
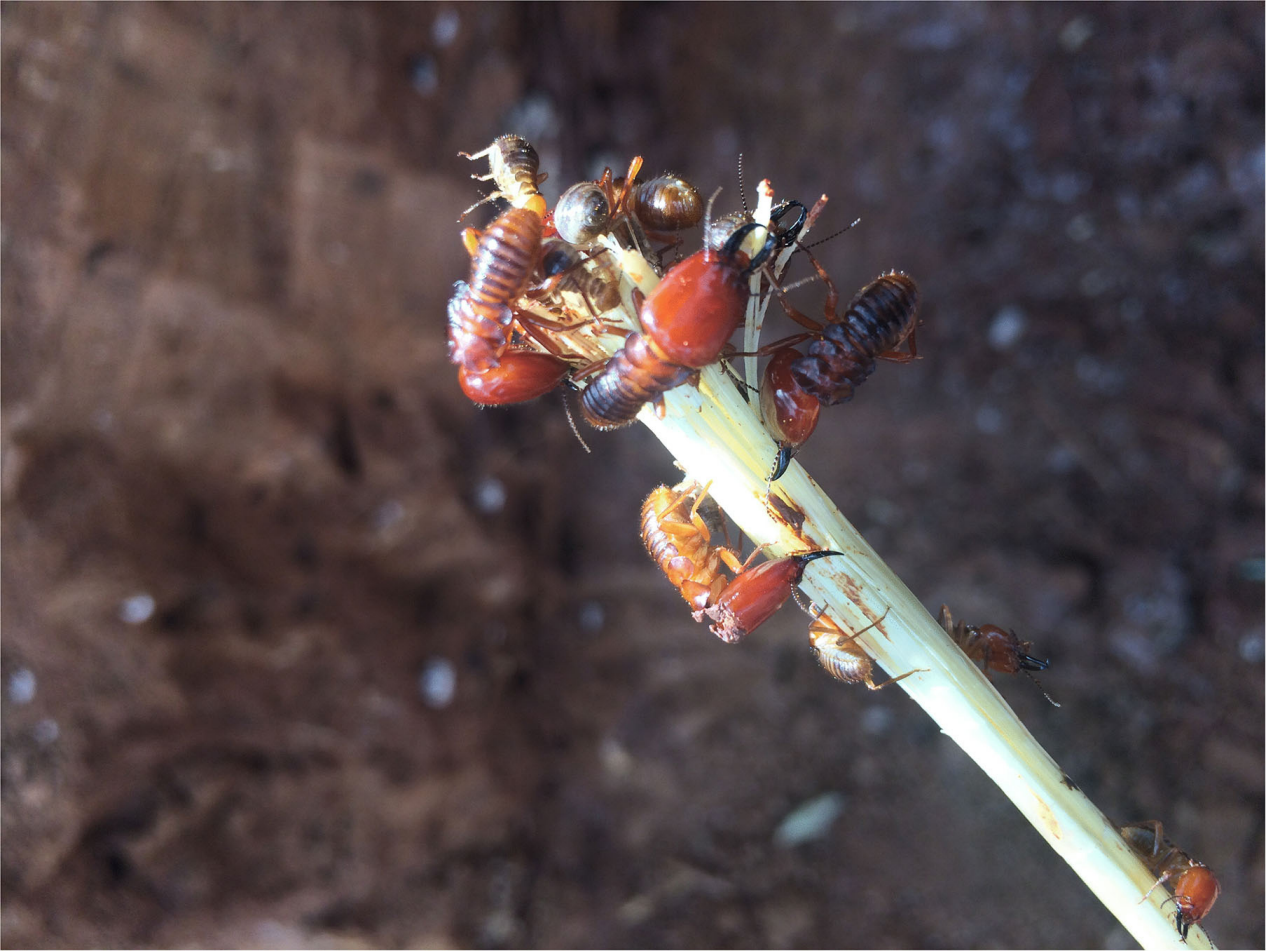
Soldier termites (*Macrotermes natalensis*) are harvested using succulent grass rods. As soldiers attack to defend the nest from perceived intrusion by the grass rod, their mandibles become entrapped on the succulent grass rod and to their doom. This method is selective to soldier termites.

**Figure 2:**
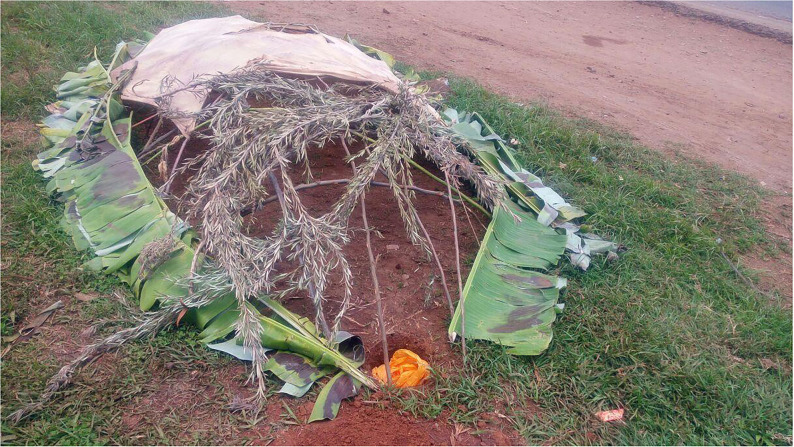
Traditional light trap used to selectively harvest the alate flying termites.

**Figure 3:**
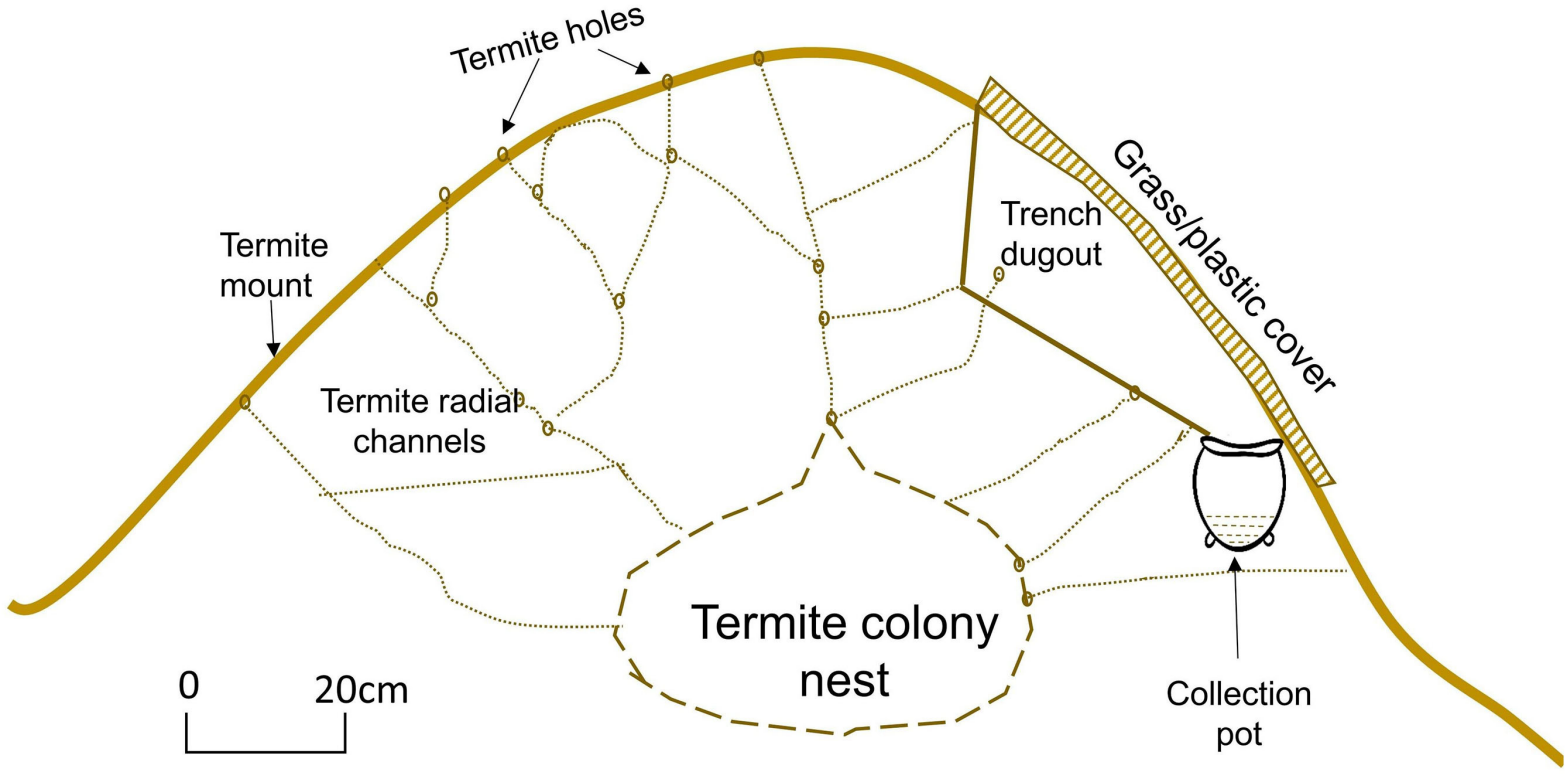
Traditional trap used to selectively harvest the alate flying termites during the rainy season.

Notable examples of sustainable approaches of selectively harvesting one form of the insect exist. For example in many parts of African communities that consume the soldier termites (such as communities in Benin, Cameroon, Kenya, Malawi, Tanzania, Zambia and Zimbabwe) specialized techniques are used to selectively capture, collect or harvest the targeted form of insects without destroying the termite colony and the breeding queen. Figure 1 illustrates the selective trapping method that only targets the soldier termites and spares the queen termite and other members of the social colony. Similarly, in order to selectively trap the flying alate form of termites from termite mounds, some communities in African countries such as Cameroon, Kenya, Malawi, Mozambique, Zambia and Zimbabwe use traditional light traps (Figure 2). Also, a gently sloping trench can be dug on the termite mound, and a collection pot placed at the lower end of the trap. The trench is then covered with grass or plastic to ensure that elates crawl into the collection pot (Figure 3). Such trapping methods specifically capture the targeted insects without destroying the rest of the colony (10).

### Evidence From Community Initiated Monitoring and Edible Insect Conservation Programmes

Social and cultural dimensions of edible insects are also at the core of stewardship in entomophagy. Duncan (35), Chavunduka (2), DeFoliart (32), and van Huis (6) articulate the various socio-cultural activities associated with insect consumption in SSA. These practices have ensured continued sustenance of entomophagy amongst the African communities invariably contributing to the conservation of insect species and their wild habitats.

In southern Africa local traditions enforce the selective harvesting of an edible stink bug (*Encosternum delegorguei*) in non-breeding sites during the insect breeding periods (25, 36, 37). This community managed system has been used for many years and is reported in folklore stories (38, 39). The present day availability of such insects as well as the habitats in which they are gathered can be attributed to these traditional practices (37). In mid-western Zambia, traditional harvesting practices are based on monitoring the abundance of caterpillars and protecting host plants and eggs against bush fires with occasional temporal restrictions in harvesting to ensure sustainability (38). In the Democratic Republic of Congo, Kinshasa (DRC), knowledge of caterpillar host plant associations enables the insect consumers to bring back young caterpillars to known host plants in forests to restore occurrences in the wild (40).

Additionally, in southern Africa, harvesting of *Henicus whellani*, an edible ground cricket, indicate a systematic community coordinated approach where crickets are gathered only after the peak mating and egg-laying period (41). Insect gatherers are cognisant of the skewed insect sex ratios that are in favor of more females in the wild compared to males (42). Indigenous insect consumers also have traditions that forbid consumption of male insects. Through song and dance, children and community members are informed about the benefits of preserving the male insects in the wild (42).

Other case studies in southern parts of Zimbabwe, Northern provinces of South Africa and eastern parts of Botswana relate to the formation of vigilant groups by indigenous communities for coordinating harvesting of mopane worms (*Gonimbrassia belina*) from natural forests in wetter regions. This has also been supported by enforcement of local laws in order to reduce deforestation of the mopane tree (*Colophospermum mopane*) and other indigenous tree species that are known to be host plants for mopane worms. By-laws for traditional harvesting of mopane worms were passed during 2020 into law by the Gwanda Municipality in Matebeleland South province of Zimbabwe.

In DRC, communities made efforts to conserve the edible caterpillar *Cirina forda* through enforcement of restrictions by traditional leaders on felling trees with a view to harvest the caterpillars. Additionally, harvesters created “reserves” or conservation areas where harvesting of the caterpillar was restricted or forbidden (5).

### Evidence From Insect Traditional Processing and Preparation Methods of Consumption

Traditional insect processing practices in Africa have always involved procedures that help to achieve food safety, reduce poisoning and improve nutritional quality of insects. Van Huis (6) and DeFoliart (32) provided extensive details on processing methods by indigenous communities in Africa. Evidence from recent documentations in parts of southern Africa (37, 42, 43), van Huis (31) indicates that several of the old traditions are still being practiced in modern insect consumption activities.

Reports of indigenous culinary stories and cuisines are scattered in gray literature throughout Africa and some are coded in songs and dances (6). Most of this evidence also exists in non-written formats but usually communicated to younger generations during traditional ceremonies (42). All these actions show deliberate effort by indigenous communities to perpetuate best practices that help to achieve nutritional benefits associated with insect consumption.

### Evidence From Possible Enabling Conducive Policies in SSA

Table 1 presents a list of 10 countries in representative regions of west, east, central, and southern Africa where at least 10 edible insect species are consumed. An examination of the policies in these countries indicated lack of any direct policy that is targeted to ensure good stewardship on the use of edible insects as food. Edible insects are mentioned in wildlife management and insect pest legislations from most African countries with minimum reference to them as food (16). However, using a framework approach by Niassy et al. (15), an assessment of the government ministries for each country shows that there is existence of opportunities to provide regulation and coordination of edible insects' use as food and feed in these countries.

**Table 1:** Sub-Saharan African countries with high diversity of edible insects consumed as food.

**Country**	**Regional geographical classification**	**Approximate number of insects species consumed**	**References**
Botswana	Southern Africa	10–25	(28, 32)
Central African Republic	Central Africa	25–50	(44)
Democratic Republic of Congo	Central Africa	>85	(28, 32, 44)
Kenya	East Africa	10–25	(28, 44)
Madagascar	Southern Africa	46–85	(28)
Malawi	Southern Africa	10–25	(28, 44)
Nigeria	West Africa	25–50	(28, 44)
South Africa	Southern Africa	>40	(32, 44)
Zambia	Southern Africa	>40	(32, 44)
Zimbabwe	Southern Africa	>40	(2, 32, 44)

For example, in all the countries listed in Table 1, the national Forestry and Wildlife Management (16) policies adequately address issues of conservation of wild habitats including natural forests. While in some countries such as Botswana, Central African Republic, DRC, South Africa and Zimbabwe, additional policies have been formulated to ensure the non-exploitative harvesting of non-timber products such as wild harvested edible insects. In such cases, the policies empower rural communities to take up stewardship on the exploitation of natural forestry resources in their vicinity and to restrict exploitation. In the end, this seems to be a sustainable approach as it involves local stakeholders. For instance, the Zimbabwean government through the National Culture Policy has provided an enabling environment by declaring the world heritage status of some forests and natural habitats of the edible insect *Encosternum delegorguei*.

As shown in Table 1, all countries have government ministries that regulate affairs on agriculture, forestry, wildlife, fisheries, health, trade and industry, culture, and heritage, environmental affairs as well as education. Given this, the policy environment in most African countries are conducive for maintaining good stewardship of edible insects as food and feed in SSA. However, this needs to be better articulated as stewardship.

## Can Current Cultural Stewardship Practices and Traditions be a Basis for Structured Formulations of Policies and Regulatory Frameworks of Edible Insects in SSA?

Although traditional methods have been effective in maintaining a level of sustainable edible insects from the wild, there are increasing calls to develop more efficient harvesting techniques to guarantee supply of insects and their products to the growing demands along the value chains (17, 18). Several weaknesses with current traditional harvesting practices have been identified which include inconsistencies in quality and quantities of insects harvested using current methods (9, 41, 45). Additionally, some traditionally based harvesting, preparation, and consumption practices have been rooted in cultural skills that can only pass on between generations through oral tradition and storytelling (2, 6). In the face of increasing shifts toward urbanization and change in demographic population toward younger people, some of the undocumented traditions have eroded and some face near extinction (19). It is anticipated that in the near future new harvesting methods should be based on the emerging science and technology tools. This could contribute toward enhancing sustainable and sufficient quantities of insects to support the expanding value chains as they arise.

There is evidence of increasing pressure on wild insect populations from human population increases, climate change, advances in science and technology, emerging markets, increasing emphasis on traditional agricultural practices such as crop and animal production. Traditional stewardship approaches of insect consumption in SSA will come under threat because of these changes. However, it is still possible to integrate the traditional steward practices within the emerging trends created by changes in environment and developments in science and technology.

Several opportunities exist if current developments can help to promote business cases associated with edible insect consumption practices in SSA. Traditional insects practices have potentail to make impact and gain financial value in the drugs and pharmaceutical industries where some of the components are required in small volumes. For example, Kinyuru et al. (8) and Adepoju and Omotayo (46) reported the potential for improvement of micronuntrient intake from winged termite *Macrotermes species*; Cito et al. (47) reported the presence of bioactive angiotensin converting inhibitory peptides in insects; Dutta et al. (48) Musundire et al. (11), Zielińska et al. (49) reported the antixodant potentail of traditionally consumed edible insect species; while Hui-Yu et al. (50) reported presence of antimicrobial peptides from insects with potential to improve human health. It is possible that some current practices can sustain exploitative drug manufacturing activities that may positively improve the livelihoods of the custodian indigenous populations. Incentives associated with direct benefits to indigenous communities may help to promote preservation of indigenous insect species and their sustainable exploitation. This has also been noted by Gondo et al. (17), Baiyegunhi and Oppong (51), and Sekonya et al. (19) on mopane worm value chains in southern Africa.

In the face of increased promotion of government conventional agricultural policies that support provision of seed, fertilizer, better livestock breeds, and expansion of land for agricultural purposes, proponents of edible insects have to develop innovations that clearly show the benefits of edible insects in order to pitch their positive aspects in the context of existing governmental policies. Promotional and awareness messages to preserve existing stewardship approaches need to be anchored and echo with institutional, national, regional and international frameworks. For example most African countries have existing national medium to long term development programmes such as Vision 2030 (by the government of Zimbabwe), and subscribe to African regional and continental programmes such as African Continental Free Trade Agreement (AfCFTA), Comprehensive Africa Agriculture Development Programme (CAADP), SADC Regional Agricultural Policy, Science, Technology and Innovation Strategy for Africa 2024 STISA, Sustainable Development Goals (SDGs) among others. Aligning edible insect stewardship approaches to these national, regional and international frameworks may help to attract the support of policy makers who may aid to advance national and regional establishment of regulatory frameworks of edible insects in SSA. A notable example of such a successful approach was the European Union (EU) policy on edible insects which approved the production and use of certain insects as food and feed in European member countries in 2019.

## Possible Change Pathway Toward Integration of Policies On Edible Insects in SSA

In SSA, despite the wide diversity and intercultural trade and exchange of edible insects such as mopane worms (*Gonimbrassia belina*), there seems to be lack of coherence among African countries on the coordinated and sustaible use of insects as valuable food and feed resources. This is in contrast to examples elsewhere in countries with non-traditional history on the use of edible insects as food and feed such as the EU, where the regulatory framework has been set at contintental rather than country level.

In the emerging context of farming insects, a number of dynamics are envisaged to arise. For example changes in agricultural practices may change as entreprenuers and farmers integrate insect farming with other traditional agricultural practices such as crop farming and large livestock rearing. We view the use of edible insects as a new sector that would require coordinated policies from African insitutions, governments as well as in regional programmes in the African Continental Free Trade Agreement (AfCFTA), Southern African Development Community (SADC), East African Community (EAC), Economic Community of West African States (ECOWAS), African Union (AU), African Phytosanitary Council of the AU. Enabling regional policy environments can assist in commoditising edible insects as food and feed. In the process this will assist in improving regional and international trade as well as facilitating exchange of biological materials or germplasm for breeding/farming purposes. However, the lack of within-country institutional and national integrated policies on edible insects is still a major impediment. There is need for lobbying for coordination of the edible insect sector emphasizing specific policies at regional and continental levels.

### Is There Evidence for National Commitment to Stewardship and Entomophagy in SSA?

Largely a number of African countries have adequate policy environments to incorporate the emerging edible insects industry, especially concerning sustainable utilization of insects in the wild. The various policies in existence give adequate ground to ensure conservation, sustainable uses as well as giving guarantee to participation by different actors and stakeholders including women and children. Through different development partners, some governments such as Botswana, Kenya and Zimbabwe, Ghana, Benin, Burkina Faso have allowed participation of smallholder women groups in edible insects' value addition. In Southern Africa, through the African Development Bank, a mopane worm canning and processing plant has been established in Zimbabwe while a canning factory has also been established in Botswana (42). To what extent these factories remain operational at full capacity remains to be seen. In Kenya, through various collaborative projects cricket rearing has been promoted in small scale households while in Benin, Burkina Faso and Ghana large scale rearing of maggots has been promoted for animal feed (52). There is evidence of a possible positive policy environment in SSA that seems to embrace commitment toward moving this sector forward from wild harvesting to insect farming.

## Future Prospects

**Figure 4:**
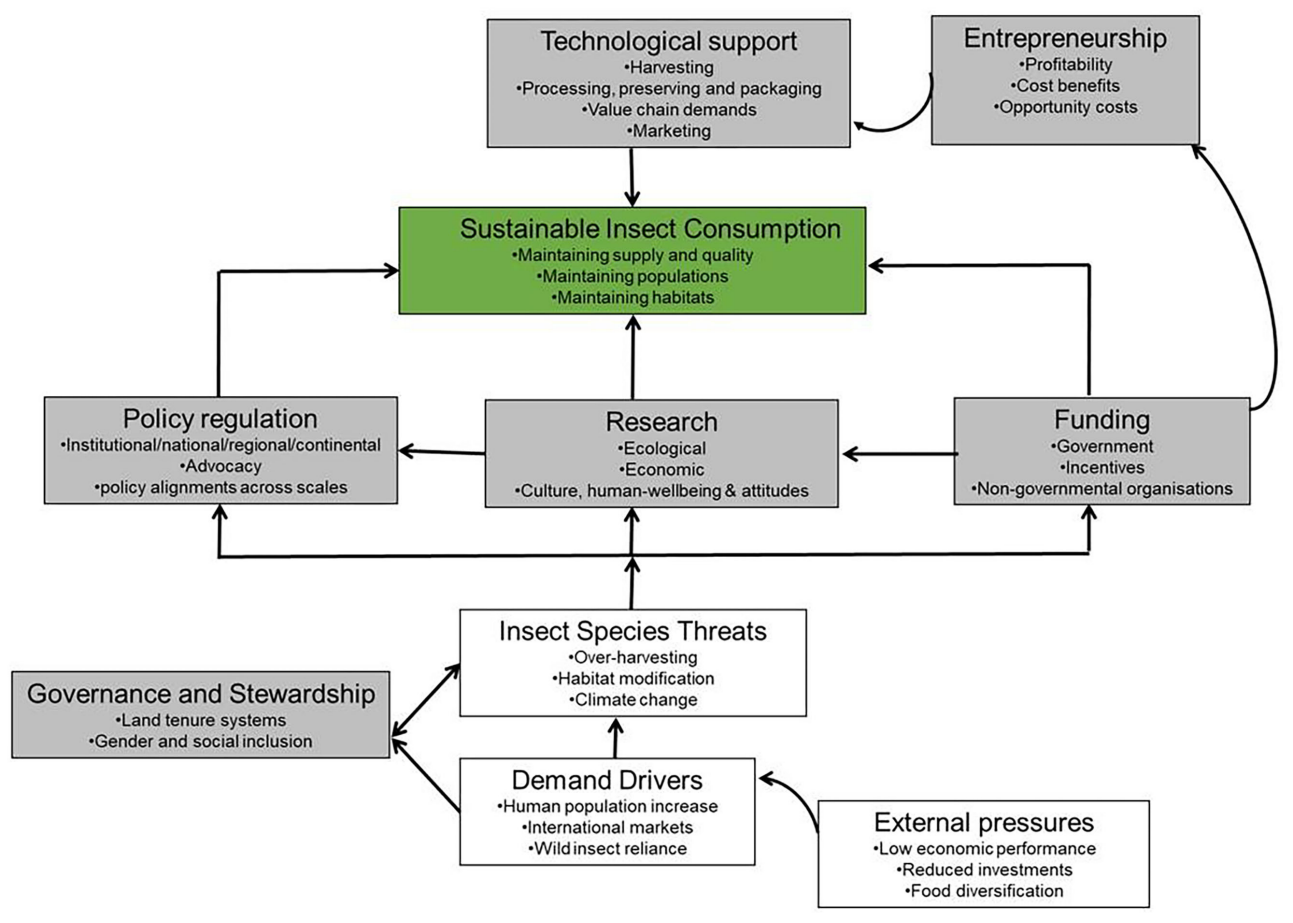
A schematic presentation of the conceptual framework of issues around promoting policy integration for sustainable edible insect consumption. The gray boxes represent sections where human interventions can be applied and the green box is the desired outcome of the process in a SSA context.

Valuable research and scientific evidence now exist in SSA that supports the importance of edible insects as nutritious food and animal feed. It is evident that the current policy environments in most SSA countries are sufficient to support sustainable and robust edible insect sectors, although there is room for regional and continental polices that govern the habitats of the edible insects such as *Gonimbrassia belina* to be developed. However, to address the dearth in edible insect specific sector policies, there is need to integrate emerging issues of this budding sector into the existing policy frameworks. Ecological, social and economic studies are therefore important to monitor the outcomes of various human interventions at various scales. Figure 4 presents a conceptual framework that could be adopted in an endeavor to support the policy framework to facilitate growth in the use of insects as food and feed in SSA. Among these could include the introduction of a national policy support on small and large scale production of some edible insects that can be used for food and feed. Support extended toward deliberate policy directives to channel a certain percentage of national budgets toward the development of an edible insect sector. Additionally, this approach could include science and technology development to support development of technologies in captive rearing as well as development of new food and feed products from insects.

While, in rural areas of most SSA countries entomophagy is embraced, national governments still shun and underestimate the contribution that this sector has on securing food security and informal income. For many women, children and marginalized communities a significant proportion of incomes derive along the insect value chain. A mind-set change needs to be cultivated amongst several stakeholders in the food sector and with potential investors. Multiple strategies need to be in place to promote and raise awareness of the value of insects as food, feed and pharmaceutical options.

## Conclusions

A number of cases in good stewardship of use of edible insects as food and feed exist in SSA. These can act as models for scaling up projects/programmes especially when it involves the sustainable utilization of edible insects in the wild. Although policies that encompass other issues such as preservation of trees, forests, water resources among others are in existence, stewardship aspects of utilization of edible insects are grossly overlooked. Stewardship of insects could easily be covered under other existing policies for most national governments in SSA. However, most government policies in SSA also lack connection to the needs of an emerging insect farming industry. This is clearly demonstrated by lack of reference of edible insects in the agricultural policies such as livestock. There is urgent need for special consideration for national support toward building and supporting the insect sector. Some entry points into strengthening communities to adopt widespread participation in the edible insect sector could be through gender, industry, trade, education and cultural ministries to promote the utilization of indigenous knowledge systems on use of edible insects as food and feed. An emerging sector as envisaged for the edible insects would require a robust regulatory framework. This review found very little evidence that demonstrates that a regulatory framework on the use of edible insects as food and feed exist for most countries in SSA.

## Author Contributions

All authors listed have made a substantial, direct and intellectual contribution to the work, and approved it for publication.

## Conflict of Interest

The authors declare that the research was conducted in the absence of any commercial or financial relationships that could be construed as a potential conflict of interest.
